# The PAediatric Risk Assessment (PARA) Mobile App to Reduce Postdischarge Child Mortality: Design, Usability, and Feasibility for Health Care Workers in Uganda

**DOI:** 10.2196/mhealth.5167

**Published:** 2016-02-15

**Authors:** Lauren Lacey English, Dustin Dunsmuir, Elias Kumbakumba, John Mark Ansermino, Charles P Larson, Richard Lester, Celestine Barigye, Andrew Ndamira, Jerome Kabakyenga, Matthew O Wiens

**Affiliations:** ^1^ School of Medicine University of North Carolina Chapel Hill, NC United States; ^2^ Pediatric Anesthesia Research Team Child and Family Research Institute University of British Columbia Vancouver, BC Canada; ^3^ Department of Pediatrics Mbarara University of Science and Technology Mbarara Uganda; ^4^ Child and Family Research Institute University of British Columbia Vancouver, BC Canada; ^5^ Division of Infectious Diseases Department of Medicine University of British Columbia Vancouver, BC Canada; ^6^ Mbarara Regional Referral Hospital Mbarara Uganda; ^7^ Maternal, Newborn and Child Health Institute Mbarara University of Science and Technology Mbarara Uganda

**Keywords:** infectious disease, postdischarge mortality, mHealth, prediction model, risk assessment, usability, Africa, resource-limited settings

## Abstract

**Background:**

Postdischarge death in children is increasingly being recognized as a major contributor to overall child mortality. The PAediatric Risk Assessment (PARA) app is an mHealth tool developed to aid health care workers in resource-limited settings such as Sub-Saharan Africa to identify pediatric patients at high risk of both in-hospital and postdischarge mortality. The intended users of the PARA app are health care workers (ie, nurses, doctors, and clinical officers) with varying levels of education and technological exposure, making testing of this clinical tool critical to successful implementation.

**Objective:**

Our aim was to summarize the usability evaluation of the PARA app among target users, which consists of assessing the ease of use, functionality, and navigation of the interfaces and then iteratively improving the design of this clinical tool.

**Methods:**

Health care workers (N=30) were recruited to participate at Mbarara Regional Referral Hospital and Holy Innocents Children’s Hospital in Mbarara, Southwestern Uganda. This usability study was conducted in two phases to allow for iterative improvement and testing of the interfaces. The PARA app was evaluated using quantitative and qualitative measures, which were compared between Phases 1 and 2 of the study. Participants were given two patient scenarios that listed hypothetical information (ie, demographic, social, and clinical data) to be entered into the app and to determine the patient’s risk of in-hospital and postdischarge mortality. Time-to-completion and user errors were recorded for each participant while using the app. A modified computer system usability questionnaire was utilized at the end of each session to elicit user satisfaction with the PARA app and obtain suggestions for future improvements.

**Results:**

The average time to complete the PARA app decreased by 30% from Phase 1 to Phase 2, following user feedback and modifications. Participants spent the longest amount of time on the oxygen saturation interface, but modifications following Phase 1 cut this time by half. The average time-to-completion (during Phase 2) for doctors/medical students was 3 minutes 56 seconds. All participants agreed they would use the PARA app if available at their health facility. Given a high PARA risk score, participants suggested several interventions that would be appropriate for the sociocultural context in southwestern Uganda, which involved strengthening discharge and referral procedures within the current health care system.

**Conclusions:**

Through feedback and modifications made during this usability study, the PARA app was developed into a user-friendly app, encompassing user expectations and culturally intuitive interfaces for users with a range of technological exposure. Doctors and medical students had shorter task completion times, though all participants reported the usefulness of this tool to improve postdischarge outcomes.

## Introduction

### Background

Infectious disease in Sub-Saharan Africa is the leading cause of child mortality, accounting for 6.3 million deaths among children under 5 years old [1]. A systematic review of pediatric postdischarge mortality found that in-hospital mortality was often exceeded by mortality rates after hospitalization. Postdischarge deaths generally occurred within several weeks of discharge and in many cases did not occur in hospitals [2]. Postdischarge death in children is increasingly being recognized as a major contributor to overall child mortality, and strategies are needed to address this issue.

One promising approach is a clinical tool to allow for early identification of at-risk patients. The PAediatric Risk Assessment (PARA) mobile app is a simple, easy-to-use mHealth tool developed to aid health care workers in resource-limited settings to identify pediatric patients at high risk of mortality. The PARA app uses prediction models to accurately predict and categorize newly admitted patients as having high or low risk of death, both in-hospital and after discharge [3,4]. This has the potential to improve in-hospital and postdischarge care provided to these children. The purpose of this manuscript is to summarize the usability evaluation conducted for the PARA app, in order to develop a user-centric design that will be accepted, useful, and usable by health care workers to ultimately reduce child mortality.

### mHealth Tools

Improved access to technology in Sub-Saharan Africa, particularly mobile technology, creates an enabling environment for mobile apps such as the PARA app. The mobile network coverage in Sub-Saharan Africa is high, with availability of third generation (3G) connections rapidly growing [5,6]. As of 2014, approximately 53% of Ugandans had a telephone connection, which has grown by 48% over the past 10 years [7]. This technological leap has opened many opportunities for mHealth initiatives in low-resource settings [5,8].

Mobile phones are currently utilized for a wide array of mHealth interventions [5]. These range from health communications to monitoring and prevention to medical decision making. There are numerous advantages to enlisting mobile technologies for public health initiatives, including their low cost, easy distribution, and wide accessibility [5,6].

### The PAediatric Risk Assessment Prediction Models

The primary PARA models [3,4] were developed to predict the likelihood of in-hospital or postdischarge mortality for children under age 5 years admitted with acute infectious diseases. The models predict risk of future mortality using demographics (time since last hospitalization), anthropometric measurements (either mid-upper arm circumference or weight for age z-score), and clinical indicators (human immunodeficiency virus status, Blantyre coma score, and oxygen saturation) collected on admission. For the prediction of postdischarge mortality, the derived models have positive and negative predictive values of 11% and 99%, respectively [4]. For the prediction of in-hospital mortality, the derived models have positive and negative predictive values of 15% and 99%, respectively [3,4]. The PARA models have particular potential for targeting high-risk children for appropriate postdischarge care. In the populations where the PARA models were derived, only 30% of children are flagged as high risk. Adoption of any postdischarge intervention in a resource-limited environment, therefore, becomes much more feasible.

### The Phone Oximeter

The Phone Oximeter is a mobile app module integrated into the PARA app that takes input from a connected noninvasive pulse oximeter. This enables users to instantaneously and accurately perform 30-second spot-check measurements of oxygen saturation (SpO_2_) when connected to a pulse oximeter [9,10]. The SpO_2_ is then recorded in the PARA app and incorporated as a variable in the PARA prediction models.

### Design Constraints and Considerations

The intended setting for the PARA app is in-patient health facilities in resource-limited countries, particularly in rural or semiurban areas. These environments pose unique design constraints, as they often do not have Internet access or consistent electricity [11]. Therefore, the app was created for offline data entry on a mobile (battery-powered) touch screen device to produce an automatic risk prediction.

The intended users of the PARA app are health care workers (ie, nurses, doctors, and clinical officers) with varying levels of education and technological exposure who work in resource-constrained settings. With these considerations, the PARA app was intentionally given a simple, compact design that utilizes routine patient data collected during pediatric admissions. It is packaged to be accessible to those with limited experience with technology, producing timely results within a busy clinical context.

### Usability Testing Objectives

This usability evaluation of the PARA app was conducted to accomplish the following objectives: (1) evaluate user performance and understand common errors made by health care workers using the PARA app, (2) iteratively improve the design, functionalities, and work flow of the PARA app, (3) elicit user satisfaction regarding the utility and design of the PARA app, and (4) understand user perceptions of the PARA app and suggested interventions using this tool.

## Methods

### System Design

At each stage of the system design process, there were multiple revisions. An initial design document was created describing the app screens necessary to collect all variables in the predictive models. From this document, the Balsamiq Mockups software (Balsamiq Studios) was then used for the generation of mockups to illustrate the intended interfaces and functionalities. The prototype PARA app was then built as script within the LNhealth platform, which was developed using Lambda Native, a cross-platform open-source development environment [12,13]. The PARA app provides many features that are not possible or much more time consuming using paper systems. These include encryption of stored data with a login page to access the app, calculation of age from date of birth, calculation of weight for age z-score, measurement of SpO_2_ with a 30-second spot-check recording, and classification of each variable contributing to the predictive model risk score as high, medium, or low contribution to mortality risk.

### Hardware Specifications

For this usability study, the PARA app was installed on the Dell Venue 7 (model 3740) device. The device was hardwired via a micro–universal serial bus (USB) connection to a mobile audio-based pulse oximeter (LionsGate Technologies). The pulse oximeter provides the photoplethysmograph waveform, the processed trend values for the SpO_2_ and heart rate, and a signal quality index (SQI).

### User Interfaces

The PARA app enables users to input, summarize, and edit select clinical information for pediatric patients admitted with an acute infectious disease. The app also allows for storage of this patient data for future review. All entries are assigned a patient ID to ensure unique identification. All interfaces contain open data entry fields or dropdown menus, with the exception of the oxygen saturation interface. The oxygen saturation interface records 30-second SpO_2_ measurements from a pulse oximeter using a color-coded SQI, time progress bar, and directional messages [7]. The postdischarge and in-hospital risk scores, displayed on the final interface, are calculated based on the specified prediction models [4]. Error messages are in place to alert the user to missing or insufficient patient data.

### Usability Evaluation Design

Early prototypes were first evaluated by study investigators and research staff in Canada and Uganda for ease of interface navigation, functionality, and basic workflow. From these initial evaluations, a testable prototype was created. This usability study was conducted in two phases to allow for iterative improvement and design of the PARA app. Ethics approval was obtained from the Mbarara University of Science and Technology (08/09-14) and the University of British Columbia (H14-01045). Participants were recruited at Mbarara Regional Referral Hospital (MRRH) and Holy Innocents Children’s Hospital (HICH), both in Mbarara, Southwestern Uganda. Health workers involved in pediatric care from MRRH and HICH were purposively sampled based on level of medical training. This may have led to participants who had a higher degree of interest in mHealth apps. An equal number of doctors/medical students and nurses/clinical officers enrolled for each phase to ensure the primary user groups were represented. No participants had used the PARA app previously. Both phases of the study had a target sample size of 15 participants (30 in total). The recommended number of participants for usability testing is at least 10 people [14]. Written informed consent was obtained from all participants. Each participant was paid an honorarium of approximately US $10.

Participants completed a short demographic questionnaire (ie, gender, occupation, age, technology use) before a facilitator guided them through the evaluation process and study instructions. The evaluation was conducted in a quiet environment with no distractions. During the evaluation, the facilitator, seated next to the participant, recorded user interaction with each interface, comments, and errors. No other individuals were present during testing. Time-on-task measurements began when the participant started the first task and ended when they completed the final task.

Following a brief introduction to the purpose of the study (see Multimedia Appendix 1), participants were given two patient scenarios with identical length and format, which listed hypothetical information (demographic, social, and clinical data) to be entered into the app (see Multimedia Appendix 2). This information reflected routine data collected upon pediatric hospital admission at MRRH and HICH. Participants were instructed to enter this information into the PARA app as though this patient was newly admitted and to determine the child’s risk of in-hospital and postdischarge mortality. The context of use during the evaluation differed from the context of expected use since the evaluation was done in a controlled environment with relevant information provided directly to the user, rather than being directly obtained from patients or their records.

During the patient scenarios, each participant was asked to think aloud, in order to assess their thought process as they used the app [15]. They were specifically instructed to comment on the layout of the app screen, the dialogue on each interface, the order of tasks, and any additional observations or opinions. Participant dialogue was recorded during each patient scenario using a digital audio recorder. At the end of each patient scenario, participants were asked to qualitatively interpret the risk score and distinguish the risk factors (listed on the summary interface) that most contributed to a high risk score.

A modified computer system usability questionnaire (CSUQ) was utilized at the end of each session to elicit participant satisfaction with the PARA app (see Multimedia Appendix 3) [16]. On the CSUQ, usability statements were evaluated on a scale from 1-7, indicating “strongly agree” to “strongly disagree.” In addition, five qualitative questions were asked to understand the practical benefits and drawbacks of incorporating the PARA app into a clinical context: (1) “What do you like most about the app?,” (2) “What do you like least about the app?,” (3) “How could the app be changed to make it easier to use?,” (4) “Please describe how you might use this app to enhance the discharge process and care after discharge,” and (5) “When would you enter patient information into the app?”

### Usability Tasks

The usability tasks (provided as a paper evaluation form) were adapted from the Pre-eclampsia Integrated Estimate of RiSk on the Move usability study and developed to encapsulate the specific functionalities of the PARA app (see Table 1) [17]. These tasked were deemed the primary essential tasks required for full use of the PARA app.

**Table 1:** Usability tasks performed.

Number	Task
1.	Log in to system
2.	Start a new patient
3.	Enter patient demographics
4.	Enter anthropometric data
5.	Measure oxygen saturation
6.	Enter clinical data
7.	Interpret summary
8.	Calculate risk score

### Metrics

The PARA app was evaluated using quantitative and qualitative measures, which were compared between Phases 1 and 2 of the study. Quantitative measures were limited to descriptive statistics. Time-to-completion was recorded for each participant and compared between patient scenarios and occupational groups.

User errors were recorded for each patient scenario and evaluated based on severity. An error was defined as any unproductive action (eg, pushing back instead of next, choosing to continue past an error message, not obtaining SpO_2_). Severity was based on the impact of errors on the achievability and specificity of the PARA risk score. Errors were categorized into navigation errors (low severity), control usage errors (medium severity), and outcome errors (high severity) (see Table 2). High severity errors prevented the risk score from being calculated or reviewed, thereby truncating the utility of the PARA app.

**Table 2:** Type of user errors.

Type of error	Definition	Common user errors
Navigation error	Misguided or unnecessary interactions often due to unfamiliarity with the app, which do not change the intended outcome (ie, accurate risk score)	Selecting the wrong button on the interface; Re-entering data unnecessarily; Starting the SpO_2_ recording with poor signal quality
Control usage error	Input of inaccurate patient or login information	Recording incorrect patient data; Entering a hypothetical SpO_2_; Entering incorrect login information
Outcome error	Risk score is not attained or incomplete data entry occurred	Bypassing error messages; Leaving data fields incomplete; Not reaching final interface to attain risk score

## Results

### Study Population

In total, 30 health care workers (ie, 11 doctors, 3 senior medical students, 14 nurses, and 2 clinical officers) participated in the PARA app usability study, with 15 male and 15 female. Participant populations between Phases 1 and 2 of testing had an equivalent number of doctors/medical students and nurses/clinical officers for each phase. More participants came from HICH (17/30, 57%) than MRRH (13/30, 43%). The majority of participants (26/30, 87%) were between 20-30 years old. All participants owned a cell phone, with the majority of doctors/medical students owning a smartphone (12/30, 40%). Few had used a tablet (6/30, 20%) or health app (7/30, 23%) previously.

### Usability Evaluation: Phase 1

For the first patient scenario, the average time-to-completion was 9 minutes 58 seconds. By the second patient scenario, the average time-to-completion dropped to 6 minutes 23 seconds. In particular, the average amount of time spent obtaining the SpO_2_ dropped from 2 minutes 23 seconds for the first patient scenario to 1 minute 28 seconds for the second patient scenario. Doctors took the least amount of time to complete the app, averaging 4 minutes 38 seconds during the second patient scenario.

In addition to time, the average number of errors dropped between the first and second patient scenario from 4.3 to 3.2 errors (see Table 3). The majority of errors were navigation errors (low severity), which decreased between the first and second patient scenarios. However, medium and high severity errors increased or stayed the same between these patient scenarios. Approximately half of the 15 participants had outcome errors, due to the SpO_2_ not being recorded. Two participants did not reach the final interface and failed to calculate the patient’s risk score.

**Table 3:** Summary of user errors by type for Phase 1.

Scenario	Navigation errors (low severity)	Control usage errors (medium severity)	Outcome errors (high severity)	Total errors (average)
1	34	20	2/7^a^	63 (4.3)
2	16	23	2/8^a^	49 (3.2)

^a^These numbers represent outcome errors leading to no risk score being generated versus outcome errors leading to incomplete data entry.

### Adaptations Between Phases 1 and 2

Based on Phase 1 results and participant feedback, modifications were made to the PARA app to decrease user errors and time-to-completion of tasks. Error-producing interfaces were simplified by adjusting the instructional dialogue, interface design, or error messages.

Errors caused by the oxygen saturation and summary interfaces were the most common issues. The oxygen saturation interface produced the most difficulties, as participants had trouble accessing the SpO_2_ screen and interpreting how to use the tablet-based system. Since the previous SpO_2_ recording was retained, some participants thought the SpO_2_ was already recorded when it was not. One participant said “So what will happen? Will [the SpO_2_ recording] stop? Will it stop or am I the one to stop it? [Sees SpO_2_ from previous recording] But I think the oxygen saturation is 93, it is just at 93. So I am moving to the next.”

Participants also had difficulty interpreting the summary of risk factors screen. When asked to identify the most important risk factors listed on the summary interface, one participant said:

I am imagining that red means danger so if it is more red, then it is contributing a lot. But you can’t tell to what degree, to what percentage. Here they are almost all the same for example, so you almost think that perhaps they all almost have perhaps the same contribution. I’m thinking like that but it’s not so clear here to tell which one or to what degree.

**Figure 1:**
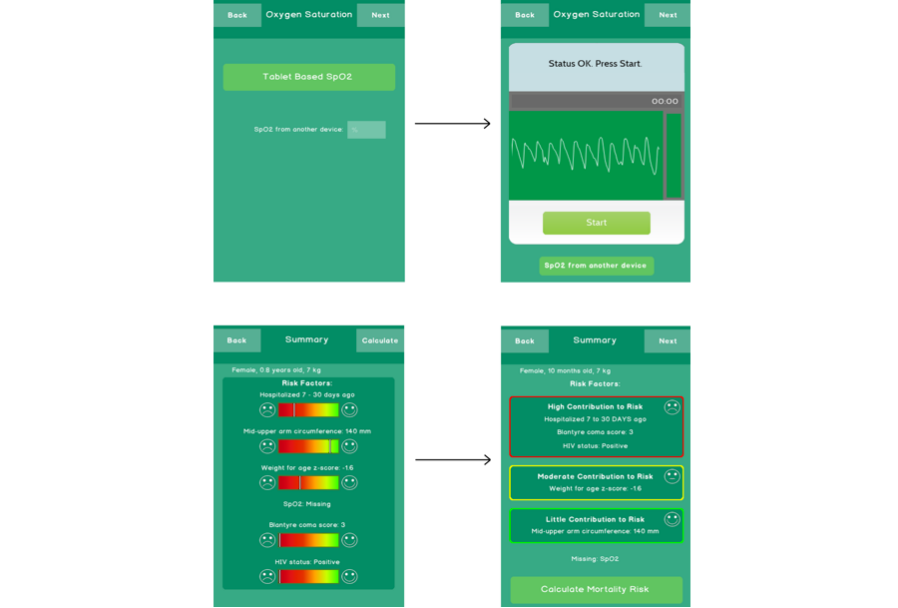
PARA app modifications to the Oxygen Saturation and Summary pages from Phase 1 to Phase 2.

Based on this user feedback, the PARA app was modified and subsequently tested during Phase 2 for improved usability (see Figure 1). Instead of showing a menu with options for measuring SpO_2_, the tablet-based SpO_2_ was changed to be the default display, with an option to enter SpO_2_ from another device at the bottom of the screen. Additional instructional messages were incorporated, indicating when to push start and when the SpO_2_ recording was complete. These were moved to the top of the screen and the current SpO_2_ and heart rate values were not displayed until the recording was started, to avoid users’ thinking they were already done. To avoid confusion, each new assessment started with a blank SpO_2_ screen instead of the previous values. For the summary interface, the risk factor scales were removed; instead risk factors were categorized into red, yellow, and green boxes and labeled as having high, medium, or low contribution to risk. A large “Calculate Risk Mortality” button was added at the bottom of the screen. This was in addition to the top right navigation button but was felt to be necessary to prevent users from stopping the app early with unattained risk scores.

### Usability Evaluation: Phase 2

For the first patient scenario, the average time-to-completion was 7 minutes 10 seconds. By the second patient scenario, the average time-to-completion dropped to 4 minutes 44 seconds. In particular, the average amount of time spent obtaining the SpO_2_ dropped from 1 minute 32 seconds for the first patient scenario to 1 minute 4 seconds for the second patient scenario. Doctors again took the least amount of time to complete the app, averaging 3 minutes 56 seconds during the second patient scenario.

The average number of errors dropped between the first and second patient scenario from 3.4 to 2.5 errors (Table 4). The majority of errors were navigation errors (low severity). These errors decreased between the first and second patient scenarios, as did control usage errors (medium severity). There were no unattained risk scores during Phase 2 of the evaluation. Though incomplete data entry occurred, it was uncommon for both patient scenarios (n=2 and n=3, respectively).

**Table 4:** Summary of user errors by type for Phase 2.

Scenario	Navigation errors (low severity)	Control usage errors (medium severity)	Outcome errors (high severity)	Total errors (average)
1	32	17	0/2^a^	51 (3.4)
2	22	12	0/3^a^	37 (2.5)

^a^These numbers represent outcome errors leading to no risk score being generated and outcome errors leading to incomplete data entry, respectively.

### Comparison of Phases 1 and 2

The average time to complete the PARA app was lower for Phase 2, following user feedback and modifications, than for Phase 1 of testing, decreasing by 30% (Table 5). Participants spent the longest amount of time on the oxygen saturation interface, but modifications following Phase 1 cut this time by half. This time savings was likely underestimated, since outcome errors (such as incomplete data entry) during Phase 1 may have artificially lowered average time-to-completion.

The average time-to-completion (during patient Scenario 2) for doctors/medical students was 4 minutes 38 seconds for Phase 1 and 3 minutes 56 seconds for Phase 2, as compared to nurses/clinical officers whose average was 7 minutes 54 seconds for Phase 1 and 5 minutes 26 seconds for Phase 2. The differences in completion times between doctors/medical students and nurses/clinical officers was statistically significant (*P*<.05) when combining Phases 1 and 2. The adjustments made to the PARA app between Phases 1 and 2 decreased the number of errors and overall time-to-completion, particularly for those with less medical education.

**Table 5:** Summary of user results for Phases 1 and 2.

	Phase 1		Phase 2	
	Scenario 1	Scenario 2	Scenario 1	Scenario 2
Average overall time	9.96 min	6.38 min	7.17 min	4.47 min
Average SpO_2_ time	2.38 min	1.47 min	1.54 min	1.07 min
Average errors	4.27 errors	3.2 errors	3.4 errors	2.5 errors

Overall, doctors/medical students had fewer user errors on patient scenarios than nurses/clinical officers (*P*<.05). During Scenario 1, doctors/medical students averaged 2.3 errors while nurses/clinical officers averaged 5.2 errors. User errors bilaterally decreased with Scenario 2, with doctors/medical students averaging 1.6 errors and nurses/clinical officers averaging 3.9 errors. Nurses/clinical officers were more likely to make outcome errors (high severity) than doctors/medical students. They accounted for all of the errors leading to no risk score (n=4), and the majority of the errors leading to incomplete data entry (n=13).

### Participant Feedback

#### Computer System Usability Questionnaire Results

CSUQ results were very low (indicating positive opinions) with no substantial differences according to phase. On a scale from 1-7 (from “strongly agreed” to “strongly disagreed”), most responses were 1, with an average score of 2.07 on all questions. Overall, people found the app easy to use and understand. All participants (n=30) strongly agreed to the statements “I liked using this app,” “The organization of information on the app screen is clear,” and “I would use this interface if it were available at my health facility.”

#### Qualitative Feedback

When asked about the positive aspects of the PARA app, participants generally commented on simplicity and utility. One participant summarized, “The app is advanced, but the interface is easy to understand.” Most reported that the app was easy to learn how to use, though some, particularly those with less medical education, requested more training. Participants felt the error messages helped guide them through the app, and many felt the PARA app was quick, as it automatically calculated the risk score preventing added burden on the health care worker. One provider said, “But the most important thing is it’s really very fast and saves time—so you are not wasting the patient’s time or your own time. That’s the most important thing, it’s really very fast.”

### Potential Applications of the PAediatric Risk Assessment App

Participants suggested several means by which the PARA app could improve patient care both in-hospital and after discharge. For children at high risk of in-hospital mortality, health care workers thought these patients would need more attention on the ward, through more frequent assessment or prioritized medication during shortages. One participant explains, “So if they are on the ward, still I can know this is a high-risk child and take extra caution in caring for this child.”

For children at high risk following discharge, participating health care workers suggested clinical and educational interventions to curb mortality (Table 6). Many recommended improved follow-up care by referring the child to a nearby health center, scheduling more frequent follow-up visits at the hospital, or calling caregivers with appointment reminders. Others suggested extending hospital stay to ensure the patient has fully recovered before discharge. Patient caregivers, particularly mothers, could be given health education at discharge, especially focused on danger signs for child mortality. This education could empower the caregiver to identify their child’s health status and respond in a timely manner.

**Table 6:** Postdischarge interventions suggested for high-risk children identified through the PARA app.

Suggested interventions	Illustrative quotes
Refer to nearby health center	“Or maybe if there is a nearby health center or what, they could also be informed about the risk of the child. They could really be followed up closely.”
Shorter review date	“I would want to see them shortly after they had been discharged and then more frequently, at least for about 3-6 months.”
Health education	“You can give advice to the patient [or caregiver]: eat well, take the medicine at the right time, make sure feed baby well, give medicine at the right time. In case of minor illness, bring child back to the hospital.”
Longer hospital stay	“I would give them a longer duration of stay in hospital but also take precaution, monitor them more closely because they are risk of dying.”
Teach parents about danger signs	“First you need to talk to the parents to make sure they understand the child is very sick and even when they improve, they still have a high risk of mortality at home, so they need to keep a close watch on the child. And in case of any symptoms, you explain to them the risk symptoms and if they feel they have identified any of them, they should call a doctor and ask if they should come [to the hospital] or if they can manage it at home.”

Though rated highly on the CSUQ, the purpose and practical application of the PARA app was sometimes unclear. The purpose of this clinical tool is to provide early indication of heightened risk of pediatric mortality. Some participants mistook the PARA app as electronic medical records, while others assumed the PARA app could be used to continually assess a patient’s progress through treatment or to make discharge decisions. These misinterpretations were summarized by one participant’s comment:

The app helps a lot because it tries to make for you a decision. It decides for you whether to discharge or not to discharge...If the patient is at high risk while at the hospital, it gives you the opportunity to discharge the patient early before the risk comes in. So I feel it can help you make a rightful decision.

## Discussion

### Principal Findings

Development of a mobile app to be utilized in a low-resource, cross-cultural setting requires iterative testing and adaptation to produce an intuitive design. Through feedback and modifications made during this usability study, the PARA app was developed into a user-friendly design, encompassing user expectations and culturally intuitive interfaces for users with a range of technological exposure. Time-to-completion and number of user errors decreased between Phases 1 and 2 of testing, following modifications made to PARA app interfaces. Overall, participants consistently reported the ability to learn and utilize the PARA app quickly and easily. The majority of errors, particularly the navigation errors, were due to unfamiliarity with apps and touch screen devices for those with limited experience.

Based on time-to-completion measurements, doctors and medical students were identified as the ideal end-users, or at least the most likely early adopters. With fairly little guidance, doctors and medical students had an intuitive grasp of the app’s purpose and functionalities. On average, nurses and clinical officers had less previous exposure to touch screen technology and therefore experienced more difficulty with the PARA app. However, all participants found the PARA app to be a useful clinical tool, agreeing they would use it if available at their health facility. The impact on workflow in a clinical environment could not be assessed in our standardized testing environment. Future research will be conducted in clinical environments utilizing actual end-users.

However, education gaps were identified during the study, which would impact future implementation and training. Some participants overestimated the scope and purpose of the PARA app, leading them to mistake the app for an electronic records system or continual assessment tool. As the PARA app is scaled in clinical settings, consideration should be given to the best way to train and educate clinicians on appropriate functionalities. The addition of a training video upon installation of the PARA app may curb misunderstanding during scale-up. Other studies have found that stakeholder collaboration, governmental support, and local adaptation are important factors to successful implementation of mHealth programs [18].

Given a high PARA risk score, participants suggested several interventions that would be appropriate for the sociocultural context in southwestern Uganda. Most suggestions centered on strengthening discharge and referral procedures within the current health care system. Participants felt that educational interventions on discharge or convenient and consistent follow-up after discharge could improve mortality outcomes for children with high PARA scores (indicating >10% risk of postdischarge mortality). Though these interventions have been studied in other contexts, little evidence exists on their effectiveness at diminishing postdischarge mortality, and more research regarding effective strategies to decrease postdischarge mortality are urgently required [2,19].

### Limitations

As this was an initial evaluation of a novel app, this usability study was not conducted in a clinical context, but instead with purposively sampled potential end-users in an artificial environment. Therefore, findings may not be generalizable to a clinical context. However, efforts were made to encompass a variety of target users, in order to understand the utility of the app from a variety of professional perspectives and technological skill levels. Further, our clinical scenarios were carefully developed to ensure a balanced and representative evaluation from both a clinical and usability/design perspective.

In addition, given varied exposure to technology, there was some degree of novelty and expectancy effects. To address these concerns, clear instructions were given to each participant on how to use a touch screen, as well as reinforcement that the only expected outcome from the study was improvement of the app. The patient scenario instructions were adjusted between Phases 1 and 2 to account for modifications made to the app. However, patient scenarios and sequence of tasks remained standardized between phases, so comparable testing conditions were preserved.

### Future Plans

Postdischarge mortality is a neglected but significant cause of child mortality in resource-constrained settings. The PARA app can begin to address this burden through its ability to quickly identify children at highest risk of death during the postdischarge period. Our research team in Uganda is currently conducting a feasibility study of a comprehensive postdischarge intervention (discharge kits) to distribute to vulnerable children at discharge. Over the next 24-36 months, our research team in Uganda will begin to integrate the PARA app with discharge kits to evaluate their effect on health seeking, hospital re-admissions, and mortality during the postdischarge period.

## Appendix

Multimedia Appendix 1 Introduction to PAediatric Risk Assessment (PARA) app.

Multimedia Appendix 2 Patient information.

Multimedia Appendix 3 Poststudy questionnaire.

## Abbreviations

CSUQ: computer system usability questionnaire
HICH: Holy Innocents Children’s Hospital
MRRH: Mbarara Regional Referral Hospital
PARA: PAediatric Risk Assessment
SpO2: oxygen saturation
SQI: signal quality index
