# Supplementary material for: The PAediatric Risk Assessment (PARA) Mobile App to Reduce Postdischarge Child Mortality: Design, Usability, and Feasibility for Health Care Workers in Uganda
Source: JMIR Mhealth Uhealth. 2016 Feb 15;4(1):e16. doi: 10.2196/mhealth.5167 (PMC4771927; doi:10.2196/mhealth.5167)
Supplement: Multimedia Appendix 1 [file mhealth_v4i1e16_app1.pdf]

Introduction to PAediatric Risk Assessment (PARA) App

Mortality following hospital discharge is an important and under-recognized contributor to overall child mortality. A recent study at Mbarara Regional Referral Hospital and Holy Innocents Children's Hospital showed that as many children died within 6 months after discharge as during their hospital stay. Children in the study were 6 months to 5 years old with a proven or suspected infectious illness. Several risk factors collected at admission were shown to be predictive of death in the hospital and following discharge. Risk prediction models were developed to predict both in-hospital and post-discharge mortality. The most important components of these models are malnutrition (small mid-upper arm circumference), HIV, low oxygen saturation, low Blantyre Coma Score, and recent hospitalization.

The PARA App is a program for smart phones or tablet computers. It uses the risk model with the 5 risk factors mentioned above to determine if a child is at high or low risk of death, both during hospitalization and following discharge. **The PARA app works by entering key patient information collected upon admission into the tablet and using this information to predict the child's risk of death.** It will tell you if the child is at high or low risk of death in hospital and at high or low risk of death after discharge.

Do you have any questions about the app or its purpose?

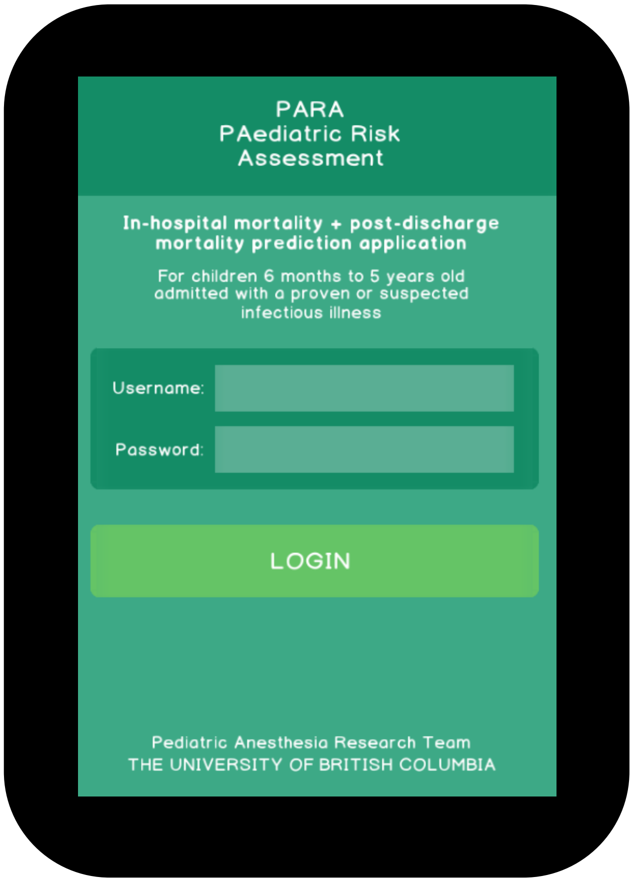

Instructions for Participants

The purpose of this study is to test and evaluate the PARA app. We want to know if it is easy to use and understand. Your honest feedback will help to improve the PARA app for its eventual use in places like Mbarara and elsewhere in East Africa.

We will give you two simulated **patient scenarios with flash cards**. The flash cards will help guide you through the PARA app, by giving patient information or instructions for each screen. **Use this patient information to fill in the blank boxes in the PARA app, and determine the child's risk of in-hospital and post-discharge mortality.**

As you go through each patient scenario:

1) We will **observe** how you interact with the app, and record these observations on our paper evaluation form.
2) We ask that you **think-aloud**. This means you say out loud whatever you are thinking, doing, and feeling, as you interact with the PARA app. We will be recording your speech throughout the experiment.

After completing two patient scenarios, you will be asked to fill out a **questionnaire** regarding the PARA app and ways to improve the usability and design.

Before we start using the PARA app, do you have any questions about the evaluation or think aloud?
