# Supplementary material for: The PAediatric Risk Assessment (PARA) Mobile App to Reduce Postdischarge Child Mortality: Design, Usability, and Feasibility for Health Care Workers in Uganda
Source: JMIR Mhealth Uhealth. 2016 Feb 15;4(1):e16. doi: 10.2196/mhealth.5167 (PMC4771927; doi:10.2196/mhealth.5167)
Supplement: Multimedia Appendix 2 [file mhealth_v4i1e16_app2.pdf]

**Patient ID: _________**

Abasa Sam is a 1 year old patient with severe pneumonia and malnutrition. Upon admission, the following patient information and vital signs are obtained:

Patient's Date of birth: March 1, 2014
Last hospitalized 3 months ago
Weight: 11 kg
MUAC: 115 mm
Blantyre Coma Scale:
- Eye movement: Fails to watch or follow
- Best motor response: Withdraws limb from painful stimulus
- Best verbal response: Moan or abnormal cry with pain

HIV Status: Positive

Measure oxygen saturation using the probe connected to the tablet.

**Instructions:**
**Enter this patient information into the PARA app to determine his risk of in hospital and post-discharge mortality.**

**Patient ID: _________**

Niwe Gift is a 3 year old patient with symptoms of sepsis. Upon admission, the following patient information and vital signs are obtained:

Patient's Date of birth: January 25, 2012
Last hospitalized 3 months ago
Weight: 16 kg
MUAC: 180 mm
Blantyre Coma Scale:
- Eye movement: Fails to watch or follow
- Best motor response: Withdraws limb from painful stimulus
- Best verbal response: No vocal response to pain

HIV Status: Negative

Measure oxygen saturation using the probe connected to the tablet.

**Instructions:**
**Enter this patient information into the PARA app to determine her risk of in hospital and post-discharge mortality.**
