# Supplementary material for: The PAediatric Risk Assessment (PARA) Mobile App to Reduce Postdischarge Child Mortality: Design, Usability, and Feasibility for Health Care Workers in Uganda
Source: JMIR Mhealth Uhealth. 2016 Feb 15;4(1):e16. doi: 10.2196/mhealth.5167 (PMC4771927; doi:10.2196/mhealth.5167)
Supplement: Multimedia Appendix 3 [file mhealth_v4i1e16_app3.pdf]

Post-Study Questionnaire

The Post-Hospital Discharge Survival Project: Improving Pediatric Care Following Hospitalization (Phase I)

Principle Investigators

Dr. Matthew Wiens	Epidemiologist	University of BC, MUST
Dr. Jerome Kabakyenga	Physician/Researcher	MUST, IMNCH
Dr. Elias Kumbakumba	Physician/Researcher	MUST

Co-Investigators (Local)

Dr. Celestine Barigye	Physician/Researcher	Ministry of Health
Dr. Andrew Ndamira	Physician/Researcher	MUST/HICH
Dr. Julius Kiwanuka	Physician/Researcher	MUST

Co-Investigator - Overseas

Dr. Charles Larson	Physician/Epidemiologist	University of BC, Canada
Dr. Mark Ansermino	Physician/Computer Engineer	University of BC, Canada
Dr. Niranjan Kissoon	Physician/Researcher	University of BC, Canada
Dr. Joel Singer	Statistician/Methodologist	University of BC, Canada

Please complete this post-study questionnaire immediately after using the PARA app. This questionnaire is based on the Computer System Usability Questionnaire (Lewis 1995).

This questionnaire gives you an opportunity to tell us your reactions to the PARA app. Your responses will help us understand what aspects of the app you are particularly concerned about and the aspects that satisfy you. Think about all the tasks that you have done with the app while you answer the questions. Please read each statement and indicate how strongly you agree or disagree with the statement by circling a number on the scale. If a statement does not apply to you, circle N/A. Please write comments to elaborate on your answers. Thank you!

Participant #:

Date:

1. Overall, I am satisfied with how easy it is to use this app.

strongly agree	1	2	3	4	5	6	7	strongly disagree	N/A

Comments:

2. It was simple to use this app.

strongly agree	1	2	3	4	5	6	7	strongly disagree	N/A

Comments:

3. This app would help me effectively complete my work.

strongly agree	1	2	3	4	5	6	7	strongly disagree	N/A

Comments:

4. I am able to use this app quickly.

strongly agree	1	2	3	4	5	6	7	strongly disagree	N/A

Comments:

5. I feel comfortable using this app.

strongly agree	1	2	3	4	5	6	7	strongly disagree	N/A

Comments:

6. It was easy to learn to use this app.

strongly agree	1	2	3	4	5	6	7	strongly disagree	N/A

Comments:

7. I believe I became productive quickly using this app.

strongly agree	1	2	3	4	5	6	7	strongly disagree	N/A

Comments:

8. Whenever I make a mistake using this app, I recover easily and quickly.

strongly agree	1	2	3	4	5	6	7	strongly disagree	N/A

Comments:

9. The information (such as on-screen messages and other documentation) provided with this app is clear.

strongly agree	1	2	3	4	5	6	7	strongly disagree	N/A

Comments:

10. The information provided for the app was easy to understand.

strongly agree	1	2	3	4	5	6	7	strongly disagree	N/A

Comments:

11. The information shown in the app screen was effective in helping me complete the tasks.

strongly agree ←→ strongly disagree

1 2 3 4 5 6 7 N/A

Comments:

12. The organization of information on the app screen is clear.

strongly agree ←→ strongly disagree

1 2 3 4 5 6 7 N/A

Comments:

13. The app is pleasant.

strongly agree ←→ strongly disagree

1 2 3 4 5 6 7 N/A

Comments:

14. I liked using this app.

strongly agree ←→ strongly disagree

1 2 3 4 5 6 7 N/A

Comments:

15. This app has all the functions and capabilities I expect it to have.

strongly agree ←→ strongly disagree

1 2 3 4 5 6 7 N/A

Comments:

16. Overall, I am satisfied with this app.

strongly agree ←→ strongly disagree N/A

1 2 3 4 5 6 7

Comments:

17. I would use this app if it were available at my health facility.

strongly agree ←→ strongly disagree N/A

1 2 3 4 5 6 7

Comments:

Qualitative Questions:

1. What do you like most about this app?

2. What do you like least about this app?

3. How could the app be changed to make it easier to use?

4. Please describe how you might use this app to enhance the discharge process and care after discharge.

5. When would you enter patient information into the app?
